# Notes on the Newer Remedies

**Published:** 1893-09

**Authors:** 


*Notes on the Newer Remedies.* By DAVID CERNA, M.D. Pp. 177. Philadelphia: W. B. SAUNDERS. 1893.

"The advance of pharmacology is such that the revised editions of those previously published, and even the new books upon the subject, become old as soon as they leave the printer's office."

The author remarks "that no barrier can obstruct the tireless march of science," and endeavouring to give the results of the checkless spirit of investigation in the field of pharmacology, he mentions a great number of drugs, mostly synthetic in character, which have been found to be more or less useful.

All the best-known modern drugs are briefly described, and their therapeutic applications are indicated, but the description is too meagre to be of much actual utility.

